# Characterization of field pea (*Pisum sativum*) resistance against *Peyronellaea pinodes* and *Didymella pinodella* that cause ascochyta blight

**DOI:** 10.3389/fpls.2022.976375

**Published:** 2022-10-24

**Authors:** Sameer Joshi, Babu Ram Pandey, Garry Rosewarne

**Affiliations:** Agriculture Victoria, Grains Innovation Park, Horsham, VIC, Australia

**Keywords:** field pea, Ascochyta blight, controlled environment, DAB staining, gene expression, resistance responses

## Abstract

Ascochyta blight is one of the most destructive diseases in field pea and is caused by either individual or combined infections by the necrotrophic pathogens *Peyronellaea pinodes*, *Didymella pinodella, Ascochyta pisi* and *Ascochyta koolunga*. Knowledge of disease epidemiology will help in understanding the resistance mechanisms, which, in turn, is beneficial in breeding for disease resistance. A pool of breeding lines and cultivars were inoculated with *P. pinodes* and *D. pinodella* to study the resistance responses and to characterize the underlying resistance reactions. In general, phenotypic analysis of controlled environment disease assays showed clear differential responses among genotypes against the two pathogens. The released variety PBA Wharton and the breeding line 11HP302-12HO-1 showed high levels of resistance against both pathogens whereas PBA Twilight and 10HP249-11HO-7 showed differential responses between the two pathogens, showing higher resistance against *D. pinodella* as compared to *P. pinodes.* OZP1604 had high infection levels against both pathogens. Histochemical analysis of leaves using diamino benzidine (DAB) showed the more resistant genotypes had lower accumulation of hydrogen peroxide compared to susceptible genotypes. The digital images of DAB staining were analyzed using ImageJ, an image analysis software. The image analysis results showed that quantification of leaf disease infection through image analysis is a useful tool in estimating the level of cell death in biotic stress studies. The qRT-PCR analysis of defense related genes showed that partially resistant genotypes had significantly higher expression of *PsOXII* and *Pshmm6* in the *P. pinodes* treated plants, whereas expression of *PsOXII*, *PsAPX1*, *PsCHS3* and *PsOPR1* increased in partially resistant plants inoculated with *D. pinodella*. The differential timing and intensity of expression of a range of genes between resistant lines challenged with the same pathogen, or challenged with different pathogens, suggests that there are multiple pathways that restrict infection in this complex pathogen-host interaction. The combination of phenotypic, histochemical and molecular approaches provide a comprehensive picture of the infection process and resistance mechanism of pea plants against these pathogens.

## Introduction

Field pea (*Pisum sativum* L.) is the most commonly grown pulse globally with important production areas including Canada, Russia, China, USA and India (FAO, 2019) (http://www.fao.org/faostat/en/#rankings/countries_by_commodity_exports). In 2020, field pea was cultivated on over 8.1 million ha with production of 14.6 million tonnes. Annual production in Australia over the past five years has been approximately 280,000 MT per year (ABARES, 2022). It is one of the most important legume crops and serves as a good source of protein for both human and animal consumption. On average, seeds contain between 15-30% protein with water-insoluble globulins and water-soluble albumins forming major fractions (Robinson et al., 2019). Furthermore, the crop plays a critical role in farming systems where it can fix atmospheric nitrogen through symbiosis with rhizobium, thus helping to reduce the use of nitrogen fertilizers (Nemecek et al., 2008; Jensen et al., 2012), as well as being a disease break crop when used in rotation with cereals and oilseeds.

Ascochyta blight, commonly known as “black spot” in Australia, is one of the most devastating diseases of field peas. It is ubiquitous in nature and has been reported in most of the field pea growing countries and can cause yield losses of up to 60% in Australia (Bretag et al., 2006). Multiple pathogens cause this disease including *Asochyta pisi* Lib. (teleom. *Didymella pisi*) (Chilvers et al., 2009), *Ascochyta pinodes* (teleomorph: *P. pinodes* (Berk. & Blox), *D. pinodella* (L.K. Jones) Morgan-Jones & K.B. Burch, and *Ascochyta koolunga* (Davidson et al., 2009) and various combinations of these can form a disease complex. In Australia, more recently *Phoma herbarum* (Li et al., 2011) and *Phoma glomerata* (Tran et al., 2014) were also reported to be part of ascochyta blight disease complex. During the 1960s, breeding focused on developing lines resistant to *A. pisi*. This likely led to *P. pinodes* becoming the most prevalent and destructive pathogen (Tivoli and Banniza, 2007).

Cooler temperatures with wet and humid conditions are most conducive to disease development (Bennet et al., 2019). These pathogens mostly infect the aerial plant parts such as leaves, stem, flower and pods. Under favorable conditions *P. pinodes* infects both seedlings and adult plants and shows symptoms of lesions on leaves and stem, foot rot and the affected seeds show shrinking and dark discoloration (Ahmed et al., 2015). *D. pinodella* causes similar symptoms to *P. pinodes*, typically being less severe on aerial parts but more severe in the roots where foot rot can extend damage to below ground plant parts (Bretag et al., 2006). *D. pinodella* survives well in warmer climates and severity of foot rot is higher in plants grown at 28° C or higher (Linford and Sprague, 1927).

Agronomic and physiological practices have been deployed in attempts to control this disease. The use of fungicides, intercropping (Fernández-Aparicio et al., 2010), reduced canopy architecture and burial of infected debris (Schoeny et al., 2008) are some of the methods to reduce the severity of infection. These methods are not ideal as the use of fungicides and burial of infected debris can harm the environment, while a reduced canopy and intercropping may lead to lower yields.

Breeding genotypes for durable resistance is the most viable option albeit limited success has been reported due to non-availability of good levels of resistance in the germplasm and lack of good screening methods (Fondevilla et al., 2008; Adhikari et al., 2014). The differential response of genotypes against *P. pinodes* identified 22 pathotypes in Canada (Xue et al., 1998), 15 in Australia (Ali et al., 1978), and 6 in Germany (Nasir and Hoppe, 1991). The resistance against ascochyta blight may be stage specific as genotypes that were resistant at the seedling stage were not always resistant when plants were mature (Ali et al., 1978). The inheritance of resistance to *D. pinodella* showed that the variety “Kinnauri” carried a single dominant resistance gene (Rastogi and Saini, 1984). Among other reports there have been several studies of incomplete resistance against *P. pinodes* in field pea germplasm albeit higher level of resistance has been detected in other *Pisum* species (Wroth, 1998; Fondevilla et al., 2005). Resistance against *P. pinodes* is a complex trait governed by quantitative trait loci (Prioul-Gervais et al., 2007; Fondevilla et al., 2008) and incorporation of multiple loci from unadapted sources brings considerable risk of transfer of unwanted alleles. The identification of multiple pathogens, pathotypes and quantitative resistance loci highlights complexities in breeding for resistance. Therefore, knowledge of specific defense responses against this pathogen can play an important role in developing strategies to improve germplasm responses to this disease.

The resistance reaction of plants against any pathogen involves a series of responses that can be either systemic or local and has been associated with cell death (Nasir et al., 1992), protein-cross linking in epidermal cell wall (Bradley et al., 1992), accumulation of hydrogen peroxide (H_2_O_2_) and peroxidase activity (Alvarez et al., 1998). The tight relationship between epidermal cell death and smaller lesion size has been demonstrated in *Pisum* spp. when inoculated with *P. pinodes* (Carrillo et al., 2013). As an antipathogen agent, H_2_O_2_ is one of the prominent reactive oxygen species (ROS) and plays a critical role in plant defense by creating a toxic environment resulting in the restriction of pathogen growth. Apart from this, H_2_O_2_ also plays a key role as a signaling molecule (Allan and Fluhr, 1997). The production of ROS can result in extensive damage to cells and may lead to cell death (Mansoor et al., 2022). This has been proposed as a mechanism for the development of a hypersensitive response upon pathogen recognition (Levine et al., 1994). The outburst of H_2_O_2_ was shown to have a critical role in stimulating salicylic acid synthesis ultimately leading to systemic acquired resistance (SAR) against *Alternaria solani* and *Verticillium dahliae* in potato (Wu et al., 1997). The interaction of H_2_O_2_ with other signaling molecules such as abscisic acid (Terzi et al., 2014) and ethylene (Yang, 2014) has been well characterized in mung bean, maize, and Arabidopsis respectively. Genes that were associated with jasmonic acid and ethylene signaling pathways were upregulated upon inoculation with *P. pinodes* in field pea (Fondevilla et al., 2011).

The regulation of defense related genes forms an integral part of the resistance mechanism and has been well characterized for various plant pathogens in field pea (Tran et al., 2018), cucumber (Pu et al., 2014), sunflower (Şestacova et al., 2016) and rice (Pan et al., 2014). The study of such genes also provides critical information about the molecules involved in plant-pathogen interactions. Previous studies have demonstrated the induction of various defense related genes such as polyphosphoinositide metabolism (Toyoda et al., 1992), phenylalanine ammonia-lyase (PAL) and chalcone synthase (CHS) (Yoshioka et al., 1992) upon infection of field pea with *P. pinodes*. The elevated transcript levels of PAL and CHS were demonstrated in the presence of elicitors from *P. pinodes* (Toyoda et al., 1993). The *Hmm6*, which encodes 6a-hydroxymaackiain methyltransferase that catalyses the terminal step in biosynthesis of pisatin, a phytoalexin from pea tissue (Wu et al., 1997), showed a 10-fold induction at 48 hours post infection (HPI) compared to 2 HPI against *Aphanomyces euteiches* (Hosseini et al., 2015). The *PsOXII* gene which encodes a peroxidase, was upregulated three-fold while *hmm6* gene showed two times higher expression in the resistant line P665 than the susceptible variety Messire upon inoculation with *P. pinodes* (Fondevilla et al., 2011). Another ROS scavenging antioxidative enzyme ascorbate peroxidase (*APX1*) was shown to have a pivotal role in scavenging H_2_O_2_ as a result of pathogen attack (Creissen et al., 1994). Fusarium head blight infection in wheat caused a rapid increase in APX activity as early as 3 HPI (Spanic et al., 2017). The oxophytodienoic acid reductase I (OPR1), one of the genes associated with jasmonic acid (JA) biosynthesis, which is involved in the plant growth and development, showed significant induction in the shoots of wheat at 24 and 72 hours post treatment with methyl jasmonate (Liu et al., 2016).

Here, we have evaluated of the disease reactions of 16 field pea genotypes originated from Australia against two pathogens, *P. pinodes* and *D. pinodella*, that cause ascochyta blight and the underlying resistance response against those pathogens. There is little reported on the resistance responses against *D. pinodella*. This work describes the underlying resistance reactions against *P. pinodes* and *D. pinodella* through detection of H_2_O_2_ (histochemical) and quantification of defense related genes through quantitative RT-PCR (molecular) approaches in a time series manner.

## Materials and methods

### Preparation of plant materials and experimental setup

Two experiments were conducted to study the defense responses of field pea genotypes against two pathogens. Experiment 1 consisted of phenotypic screening of 16 genotypes that were chosen from previous knowledge of their reactions to infection with *P. pinodes*. Experiment 2 was conducted for the histochemical and molecular characterization of four resistant and susceptible genotypes.

In experiment 1, the genotypes were assessed for their reactions to *P. pinodes* and *D. pinodella* in the CE assay. Based on the disease scores of experiment 1 the four best and worst performing genotypes in terms of disease scores were selected for a histochemical analysis, and the top and bottom two genotypes in terms of scores were selected for molecular characterization against the same pathogens in experiment 2. The list of genotypes used in experiment 1 and experiment 2 are presented in **Tables 1**, **2** respectively. Pots with a diameter of 13.5 cm and a depth of 13.5 cm were filled with legume mix (Biogro, SA, Australia) and planted with three seeds per pot of each genotype. In the experiment 2, pots with a diameter of 7 cm and a depth of 16 cm were filled with the same potting mix and planted with one seed per pot. The leaf samples were harvested at four time points for molecular characterization.

**Table 1:** The field pea genotypes used in the controlled environment experiment 1.

Sl. Nr	Genotypes	Status
**1**	05H161-06HOS2005-BOG09-2	Breeding line
**2**	PBA Butler	Cultivar
**3**	OZP1305	Breeding line
**4**	PBA Oura	Cultivar
**5**	OZP1408	Breeding line
**6**	09HP216-10HO2-3	Breeding line
**7**	10HP249-11HO-7	Breeding line
**8**	11HP028-12HO-3	Breeding line
**9**	11HP160-12HO-1	Breeding line
**10**	11HP302-12HO-1	Breeding line
**11**	PBA Wharton	Cultivar
**12**	11HP420-12HO-13	Breeding line
**13**	Kaspa	Cultivar
**14**	PBA Twilight	Cultivar
**15**	OZP1604	Breeding line
**16**	WAPEA2211	Breeding line

**Table 2:** The field pea genotypes used in the controlled environment experiment 2.

Pathogen	Genotype	Group	Pathogen	Genotype	Group
***Peyronellaea pinodes***	05H161-06HOS2005-BOG09-2	Resistant	*Didymella pinodella*	10HP249-11HO-7	Resistant
***Peyronellaea. pinodes***	09HP216-10HO2-3	Resistant	*Didymella pinodella*	11HP302-12HO-1	Resistant
***Peyronellaea. pinodes***	11HP302-12HO-1	Resistant	*Didymella pinodella*	PBA Twilight	Resistant
***Peyronellaea pinodes***	PBA Wharton	Resistant	*Didymella pinodella*	PBA Wharton	Resistant
***Peyronellaea pinodes***	10HP249-11HO-7	Susceptible	*Didymella pinodella*	09HP216-10HO2-3	Susceptible
***Peyronellaea pinodes***	11HP160-12HO-1	Susceptible	*Didymella pinodella*	OZP1604	Susceptible
***Peyronellaea pinodes***	OZP1604	Susceptible	*Didymella pinodella*	PBA Oura	Susceptible
***Peyronellaea pinodes***	PBA Twilight	Susceptible	*Didymella pinodella*	WAPEA2211	Susceptible

Seedlings were grown in the CE with a day/night temperature of 24°/15° C with a 16:8 h light: dark cycle. When the seedlings were at the 3-4 node stage, they were transferred to a growth chamber with a temperature of 15° C and 12 h each light and dark period for inoculation. The inoculation was carried out in custom made translucent tents that had pipe fittings to allow misting from a humidifier. The plants were inoculated with either the *P. pinodes* or *D. pinodella* pathogens. The plants were transferred to the CE 24 h prior to the inoculation to acclimatize to the growth conditions. The experimental setup in the CE is presented in **Supplementary Figure S1**.

### Preparation of inoculum and inoculation

Isolates of *P. pinodes* (ID: Twilight) and *D. pinodella* (ID: MPA) were grown on Potato Dextrose Agar plates for 2-3 weeks. Conidia were harvested by flooding the plates with sterile distilled water and gently rubbing the surface of the agar with a glass spreader to loosen conidia. A hemocytometer was used to determine the spore count and the concentration was adjusted to 1 x 10^5^ (Hwang et al., 2006) conidia per ml in sterile distilled water. Pulse penetrant (Nufarm, Victoria, Australia), a surfactant, was added at 0.06% to the prepared inoculum just before inoculation. Plants of the control treatment were sprayed with sterile distilled water mixed with 0.06% pulse penetrant. In experiment 1, the three plants in each of four replicated pots of each genotype that were to be inoculated were sprayed evenly with the inoculum and tents were closed to maintain humidity. All inoculated plants were scored seven days post inoculation using a continuous scale of 0 to 9 as described by Xu et al. (1996), where 0 represented no infection and a score of 9 represented 90-100% infection. The best performing genotypes from experiment 1 were selected to study the underlying resistance response against *P. pinodes* and *D. pinodella* in the time series of experiment 2, where one plant in each of three replicated pots of each genotype was evenly sprayed with the inoculum.

### Detection of hydrogen peroxide localization using DAB staining

The accumulation of H_2_O_2_, a ROS, was detected through diaminobenzidine (DAB) staining. The methodology described here for *in situ* detection of H_2_O_2_ was adapted from Daudi and O'Brien (2012). In brief, at 72 and 96 HPI samples were taken per pathogen, genotype and replicate and placed in Petri dishes. DAB staining solution (1mg/ml) was prepared in sterile distilled water at pH 3. The solution was covered with aluminium foil due to its sensitivity to light. An aliquot of 25 µl of Tween 20 (0.05 v/v) together with 2.5 ml of 200 mM sodium phosphate solution were added to DAB to prepare 10 mM sodium phosphate DAB staining solution. The staining solution was applied to the Petri dish containing the leaf, ensuring that the leaf was completely immersed. The plates were placed in an opaque box and gently agitated on a shaker for 4 h at 100 rpm. Following incubation, the DAB solution was replaced by bleaching solution (ethanol:acetic acid:glycerol = 3:1:1) and placed in the water bath (90-95° C) for 15 min. This helps to remove chlorophyll but retains the brown precipitation caused by DAB reacting with H_2_O_2._ After the required duration of the heat treatment, the bleaching solution was replaced by fresh bleaching solution and incubated at room temperature for 30 min. Samples were stored at 4° C overnight. The leaves were visualized the following day for DAB staining and digital photographs of the leaves were acquired.

### Quantification of leaf disease infection through DAB staining

The digital images of DAB stained leaves were analyzed using ImageJ software (version 1.53c, https://imagej.nih.gov/ij/). The polygon selection tool was used to select the edges of the image followed by an inverse of the image. This selects the whole region of the image except the inversed image. The area outside of the leaf in the inversed image was filled to remove from subsequent analysis. A color threshold was applied to select the disease affected part by using HSB (Hue*Saturation*Brightness) color space. The resulting image was then converted to binary, and a mask was generated. The mask outlines the region of interest (ROI). The ROI manager tool was used to extract the pixel numbers of the disease affected part. Similarly, the total pixel numbers of the whole leaf without applying threshold were estimated. The percentage of the disease affected part was calculated using the pixel numbers of disease affected part and whole leaf.

### Sample collection, extraction of RNA and synthesis of cDNA

The leaves of pea plants that were inoculated with *P. pinodes* and *D. pinodella* were harvested at 0, 24, 72 and 96 hours post infection (HPI). For each of the inoculated treatments three biological replicates were harvested in aluminium foil, snap frozen in liquid nitrogen and stored at -80° C until further use. The bench tops, glassware, pestles, and mortars were treated with RNasZAP™ (Sigma Aldrich, St. Louis, MO, USA). Total RNA was extracted from 100 mg of each of the treated leaves using Spectrum™ Plant Total RNA kit (Sigma Aldrich, St. Louis, MO, USA) as per manufacturer’s protocol. The concentration of RNA was determined by a Nanodrop 2000 spectrophotometer (Thermal co., USA) and the integrity and quality were confirmed by loading on 1% agarose gel stained with SYBR safe. (Thermo Fisher Scientific, Carlsbad, CA). Contaminating DNA was removed by digesting the RNA sample with DNase I (RNase-free) (New England Biolabs, Ipswich, MA). The DNase I treated RNA and cDNA of random samples were confirmed for the absence of genomic DNA by performing a PCR with *PsGAPDH* primers that amplify an intron-exon-intron sequence of field pea glyceraldehyde-3-phosphate dehydrogenase (GAPDH) gene (Die et al., 2010; Fondevilla et al., 2011; Tran et al., 2018). The *PsHistone3* primers were used as PCR control along with *PsGAPDH* primers. A 500 ng aliquot of total RNA was used to synthesize cDNA using the Lunascript RT supermix system (New England Biolabs, Ipswich, MA).

### Gene expression analysis using qRT-PCR

The qRT-PCR was performed on three biological and three technical replicates to study the real time expression of defense related genes. The CFX384 touch real-time PCR detection system (Bio-Rad Laboratories, Inc., Hercules, CA, USA) was used to perform the reaction. The 10 µl qRT-PCR reaction mix consisted of 5 µl of 2X Luna universal qPCR master mix (New England Biolabs, Ipswich, MA), 1 µl (10 µM) of each of the forward and reverse primers, 1 µl (1 µg) cDNA and remaining of nuclease free water. The primers used in the qRT-PCR are provided in the **Table 3**. The standard curve for each qRT-PCR primer pairs was generated by plotting logarithm of four step 10 fold dilutions (10^0^, 10^-1^, 10^-2^ and 10^-3^) of starting pooled cDNA quantity and threshold cycle (Ct) values. The qRT-PCR reaction was performed using the following conditions: 95°C for 1 min, 39 two-step cycles each at 95°C for 15 s and 60°C for 30 s, with a plate read after each cycle and a final melting curve of 60–95°C for 5 s with an increment of 0.5°C per melt curve temperature and a plate read after each temperature step. The slope and the R^2^ values of the standard curve were calculated. The efficiency (E) was calculated using the formula E = (10^(-1/slope)^) -1. The qRT-PCR reactions were carried out in triplicate and included a no template control. The ΔCt of non-inoculated and inoculated samples for each GOI at each time points were used to calculate the ratio of relative mRNA levels using the formula as proposed by (Pfaffl, 2001),


$$R=\frac{{\left(E_{target}\right)}^{\Delta Ct_{target\left(control-sample\right)}}}{{\left(\begin{array}{c} E_{\;reference} \end{array}\right)}^{\Delta Ct_{reference\left(control-sample\right)}}}$$

**Table 3:** The RT-qPCR primers of defense related and reference genes used in this study.

	Name	Primer sequence	Amplicon (bp)	Reference
**1**	*PsOX11-F*	CTTGGAGGACCCACATGGAT	61	(Fondevilla et al., 2011)
	*PsOX11-R*	TTTGGCTTGCTGTTCTTGCA		
**2**	*PsApx1-F*	GGCACTCTGCTGGTACTTTTG	72	(Fondevilla et al., 2014)
	*PsApx1-R*	CGGCTTGGTGCTTAATTGTT		
**3**	*Pshmm6-F*	TTTGAACTTTGTTGGTGGAGATATG	80	(Fondevilla et al., 2011)
	*Pshmm6-R*	AATCATGCAGAACCCACTTGAGT		
**4**	*PsCHS3-F*	CCAAACTGTTAGGTCTTCGTCCAT	65	(Fondevilla et al., 2014)
	*PsCHS3-R*	GGCAAAACACCCTTGTTGGT		
**5**	*PsOPR1-F*	AAGTGAATGACAGAACCGATGA	60	(Fondevilla et al., 2011)
	*PsOPR1-R*	ATGGAAACCGACAGCGATT		
**6**	*PsHistone3-F*	GGAAGTATCAGAAGAGCACAGA	182	(Knopkiewicz and Wojtaszek, 2019)
	*PsHistone3-R*	AATGGCACAAAGGTTGGTATC		
**7**	*PsGAPDH-F*	GTGGTCTCCACTGACTTTATTGGT	156	(Die et al., 2010)
	*PsGAPDH-R*	TTCCTGCCTTGGCATCAAA		

1:5, defense related genes; 6:7, reference genes; F, Forward; R, Reverse; bp, base pairs.

where relative expression ratio, *E_targert_* and *E*_*reference*_  are qRT-PCR efficiencies of the target and reference genes respectively, ***ΔCt*_*target*_** is the difference in Ct values of control and sample for GOI and ***ΔCt*_*reference*_** is the difference in Ct values of control and sample for reference gene. The geometric mean of the expression levels *PsHistone3*, an endogenous reference gene was used to calculate the normalization index (Fondevilla et al., 2011; Tran et al., 2018).

### Statistical analyses

The two experiments were performed using a randomized complete block design. Experiment 1 and experiment 2 were conducted with four and three replicates of each genotype, respectively. The disease severity of genotypes was scored in the experiment 1 and the severity of disease in each genotype in the experiment 2 was assessed using DAB staining. The data was analyzed using R statistical software (https://cran.r-project.org). To test the consistency of performance of the genotypes from two experiments, the association between the leaf damage digital area of experiment 2 and disease scores of experiment 1, was studied using Pearson’s correlation (r). The disease scores and qRT-PCR fold change were analyzed by non-parametric Kruskal-Wallis rank-sum test and multiple comparisons among genotypes were performed using False Discovery Rate correction. The pathogen aggressiveness was determined by analyzing the disease scores of each pathogen using Wilcoxon-Mann-Whitney test.

## Results

### Performance of genotypes in the disease assay

**Figure 1:**
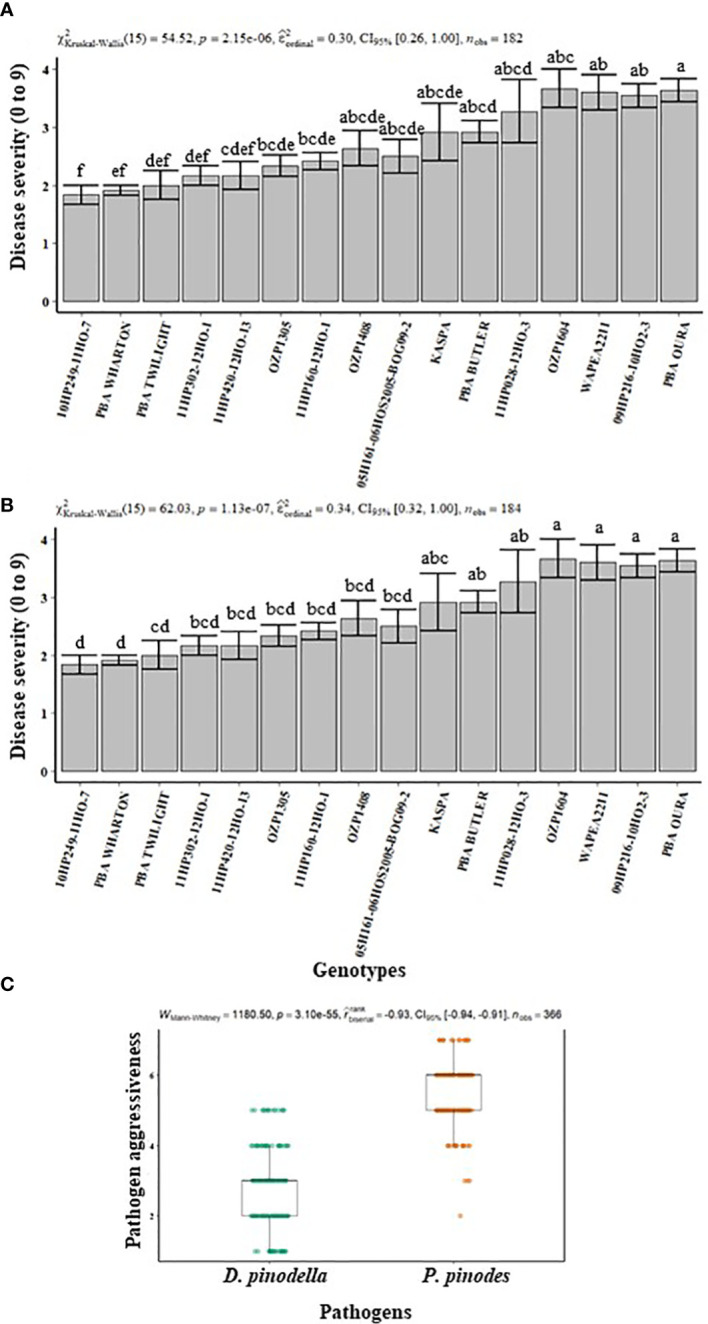
Disease severity **(A, B)** and pathogen aggressiveness **(C)** observed in field pea plants inoculated with *Peyronellaea pinodes* and *Didymella pinodella* at controlled environment facility in Horsham. The data are presented as mean ± SEM. Different letters on each bar signifies statistical significance among genotypes at *P* < 0.05 level for *P. pinodes* and *D*. *pinodella*.

In experiment 1, 16 genotypes were assessed for their performance against infection by *P. pinodes* and *D. pinodella.* In general, there was a differential response between genotypes for disease severity for both pathogens. The severity of infection ranged from 2 to 7 for *P. pinodes* whereas for *D. pinodella* the range was 1 to 5. Among the plants inoculated with *P. pinodes*, along with the released variety PBA Wharton, the breeding lines 11HP302-12HO-1, 05H161-06HOS2005-BOG09-2 and 09HP216-10HO2-3 showed significantly lower infection compared to the susceptible genotypes Kaspa, PBA Twilight, 11HP160-12HO-1 and OZP1604 (**Figure 1A**). In contrast the breeding lines 10HP-249-11HO-7 and 11HP302-12HO-1 showed significantly reduced infection compared to OZP1604, WAPEA2211 and PBA Oura upon inoculation with *D. pinodella* (**Figure 1B**). Additionally, the genotypes showed varied disease severity in response to the two pathogens. The genotype 10HP249-11HO-7 had lowest disease score of 1 against *D. pinodella* while the same genotype showed relatively higher disease score of 7 against *P. pinodes* (**Figures 1A, B**). The genotype OZP1305 had a low disease score of 1 against *D. pinodella* while moderately high disease score of 5 was observed against *P. pinodes*. Based on the differential responses of the genotypes against both pathogens, the best performing four resistant and four susceptible genotypes were selected to characterize the resistance against *P. pinodes* and *D. pinodella*. Overall, the disease analysis showed that *P. pinodes* infection occurred earlier and was more aggressive than *D. pinodella* with median scores of 6 and 3 respectively (**Figure 1C**).

### Detection of H_2_O_2_ localization and quantification of leaf damaged digital area

**Figure 2:**
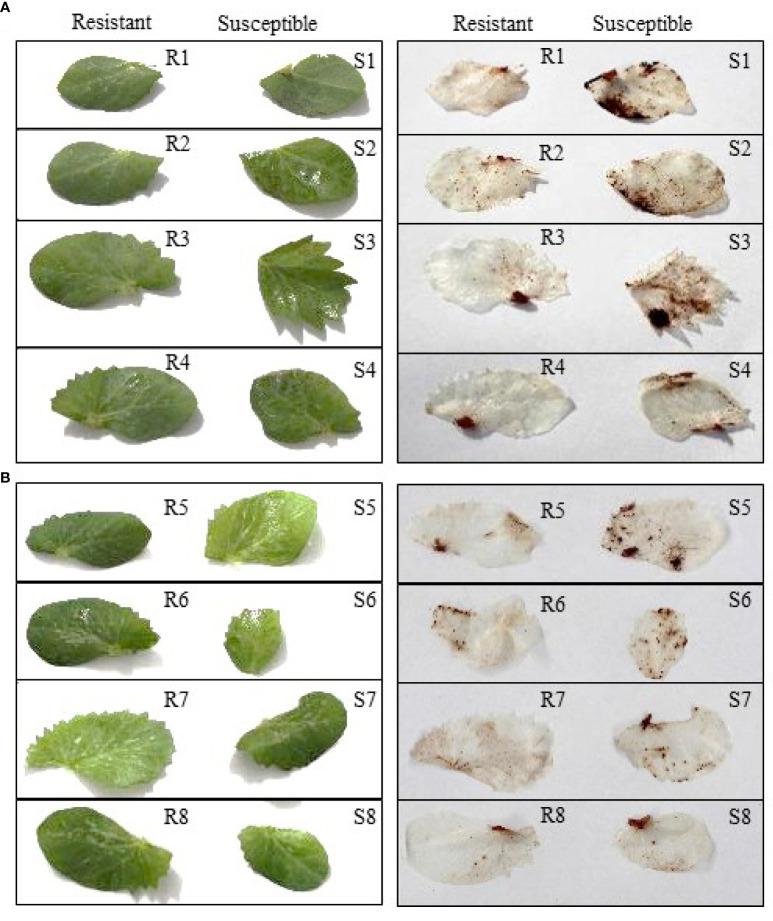
Detection of hydrogen peroxide through di-amino benzidine (DAB) staining in field pea leaves, visualized at 96 hours post inoculation with *Peyronellaea pinodes* **(A)** and *Didymella pinodella* **(B)**. R1, PBA Wharton; R2, 11HP302-12HO-1; R3, 05H161-06HOS2005-BOG09-2; R4 09HP216-10HO2-3; S1, OZP1604; S2, 11HP160-12HO-1; S3, PBA TWILIGHT; S4, 10HP249-11HO-7; R1-4 partially resistant and S1-4, susceptible to *P. pinodes.* R5, 10HP249-11HO-7; R6, PBA WHARTON; R7, PBA TWILIGHT; R8, 11HP420-12HO-13; S5, WAPEA2211; S6, OZP1604; S7, PBA OURA; S8, 09HP216-10HO2-3; R5-8 partially resistant and S5-8 susceptible to *D. pinodella*.

The field pea leaves inoculated separately with *P. pinodes* and *D. pinodella* showed accumulation of H_2_O_2_ which was observed as dark-brown precipitates due to oxidation of DAB by H_2_O_2_ and peroxidase (**Figure 2**). H_2_O_2_ accumulation was observed in response to infection as early as 72 HPI but was more evident at 96 HPI. Higher level of H_2_O_2_ was observed in the susceptible genotypes compared to their partially resistant counterparts. The varying amount of H_2_O_2_ accumulation in the partially resistant genotypes demonstrated the differential response against *P. pinodes* (**Figure 2A**). The accumulation of H_2_O_2_ was evident wherever black spot symptoms were observed. Moreover, a greater level of H_2_O_2_ accumulation was observed in the leaves inoculated with *P. pinodes* compared to *D. pinodella* (**Figures 2A, B**) which verified the aggressiveness of pathogen. The results of DAB staining corroborate with the scores obtained by the disease assay phenotyping.

**Figure 3:**
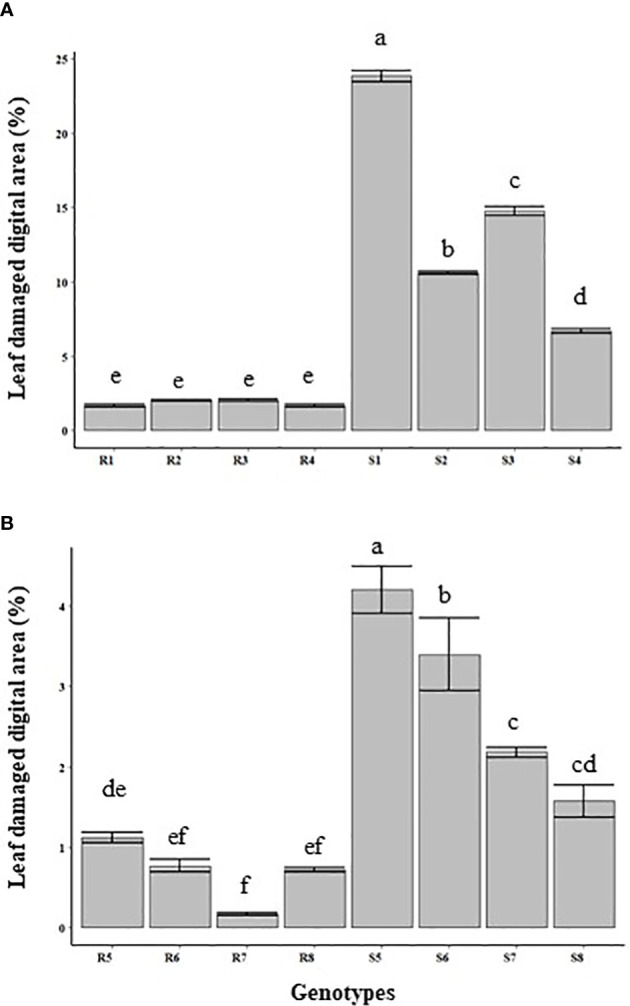
Quantification of the leaf area affected at 96 hours post inoculation with *Peyronellaea pinodes* **(A)** and *Didymella pinodella* **(B)**. The data are presented as mean ± SEM. Different letters on each bar signifies statistical significance among genotypes at *P* < 0.05 level for *P. pinodes* and *D*. *pinodella.* R1, PBA Wharton; R2, 11HP302-12HO-1; R3, 05H161-06HOS2005-BOG09-2; R4 09HP216-10HO2-3; S1, OZP1604; S2, 11HP160-12HO-1; S3, PBA TWILIGHT; S4, 10HP249-11HO-7; R1-4 partially resistant and S1-4, susceptible to *P. pinodes.* R5, 10HP249-11HO-7; R6, PBA WHARTON; R7, PBA TWILIGHT; R8, 11HP420-12HO-13; S5, WAPEA2211; S6, OZP1604; S7, PBA OURA; S8, 09HP216-10HO2-3; R5-8 partially resistant and S5-8 susceptible to *D*. *pinodella*.

**Figure 4:**
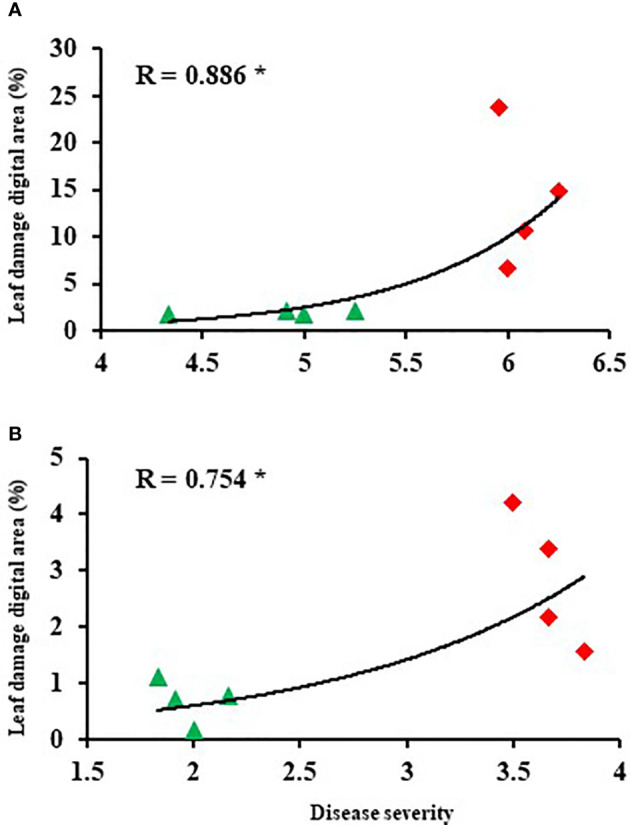
Correlations between disease score and leaf damage digital area at 96 hours post inoculation with *Peyronellaea pinodes* **(A)** and *Didymella pinodella* **(B)**. The asterisks show the significance level (* p < 0.05).

The damaged leaf area (%) was quantified through image analysis. The extent of leaf damage recorded in the susceptible genotypes was in the range of 6.7% to 23.8% of total leaf area in the leaves inoculated with *P. pinodes*, and 1.5% to 4.2% in leaves inoculated with *D. pinodella* (**Figures 3A, B**). In partially resistant genotypes, the infection was at a minimal level and in the range of 1.6 to 2% in the leaves inoculated with *P. pinodes* and 0.1 to 1.1% in case of *D. pinodella* inoculated leaves. There was a close association between the leaf damage digital area of experiment 2 and disease severity of experiment 1 with a correlation co-efficient of r = 0.89 when inoculated with *P. pinodes* and r = 0.75 when inoculated with *D. pinodella* (**Figures 4A, B**).

### Induction of defense related gene expression in the leaves

**Figure 5:**
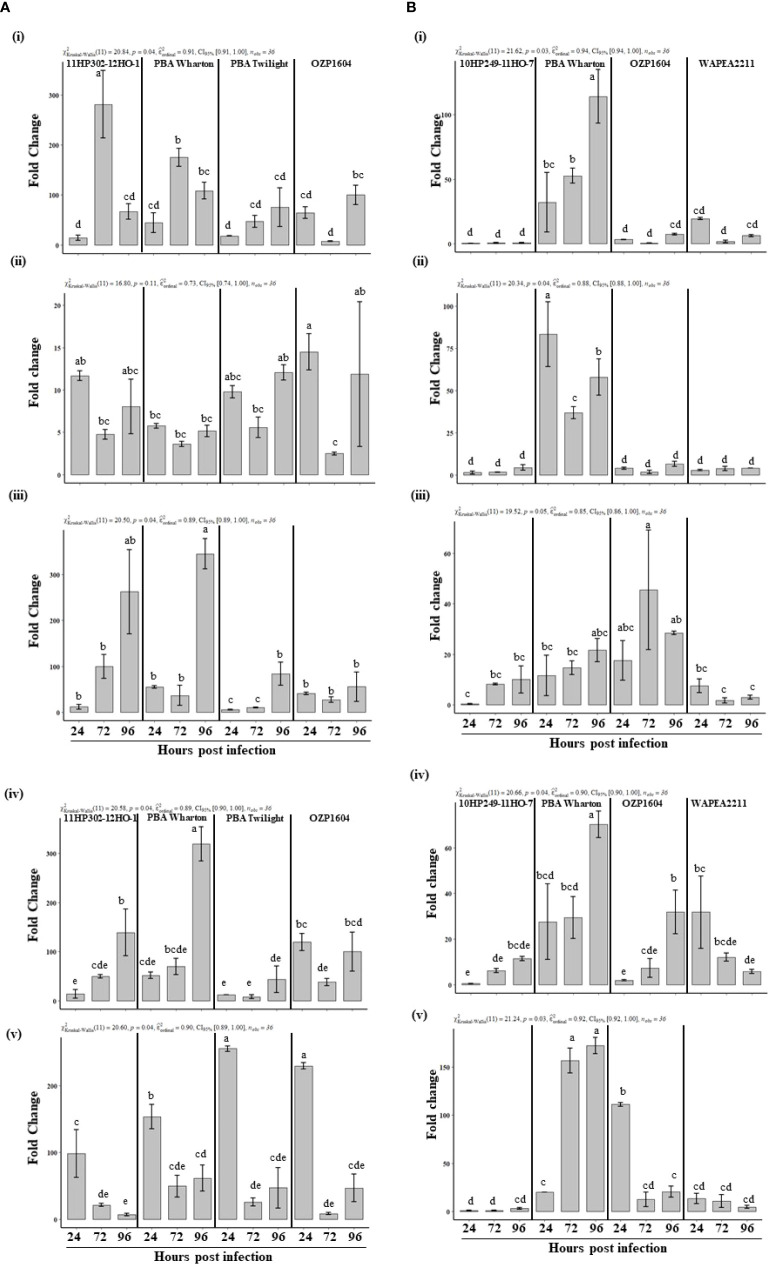
**(A)** Relative expression of defense related genes, *PsOXII* **(i)**, *PsAPX1* **(ii)**, *Pshmm6* **(iii)**, *PsCHS3* **(iv)**, *PsOPR1* **(v)** at 24, 72 and 96 hours post inoculation with *Peyronellaea pinodes.* The mRNA levels of defense related genes were normalized against reference gene *PsHistone3*. The data are presented as mean ± SEM. Different letters on each bar signifies statistical significance among genotypes at *P* < 0.05 level for *P. pinodes.* **(B)** Relative expression of defense related genes, *PsOXII* **(i)**, *PsAPX1* **(ii)**, *Pshmm6* **(iii)**, *PsCHS3* **(iv)**, *PsOPR1* **(v)** at 24, 72 and 96 hours post inoculation with *Didymella pinodella.* The mRNA levels of defense related genes were normalized against reference gene *PsHistone3*. The data are presented as mean ± SEM. Different letters on each bar signifies statistical significance among genotypes at *P* < 0.05 level for *D. pinodella*.

The timing and degree of expression of defense related genes in leaves were investigated in field pea genotypes that had varying levels of resistance. The slopes derived from the standard curves of all the defense related and reference genes were found to range from 89.9% (*PsCHS3*) to 105.6% (*PsOPR1*) (**Supplementary Figure S2**). The Histone 3 (*PsHistone3)* gene was used as an internal control to normalize the expression of defense related genes. This gene had stable expression throughout the time course of the experiment. Among five defense related genes, peroxidase (*PsOX11*), showed an earlier upregulation while both 6a-hydroxymaachiain methyltransferase (*Pshmm6*) and chalcone synthase (*PsCHS3*) were upregulated and peaked at later time point, in the partially resistant genotypes. upon inoculation with *P. pinodes* (**Figure 5A**). Although the susceptible genotype OZP1604 showed an induction of *PsOX11* and *PsCHS3*, it was significantly lower than 11HP302-12HO-1 and PBA Wharton respectively. The gene *PsOX11* showed a gradual induction, reaching a peak at 72 HPI in the partially resistant genotypes 11HP302-12HO-1 (280 fold) and PBA Wharton (175 fold), followed by a reduced induction level at 96 HPI. In the partially resistant genotypes 11HP302-12HO-1 and PBA Wharton, the relative mRNA levels of 262 and 345 fold changes were observed for the gene *Pshmm6*, while an incremental fold change was observed from 24 to 72 HPI and peaking expression of 128 and 319 at 96 HPI was observed for the gene *PsCHS3.* A distinguishable level of induction in the expression of *PsAPX1* was observed in the susceptible genotypes PBA Twilight and OZP1604. Interestingly there was a rapid induction of *PsOPR1* that peaked at 24 HPI and was observed in both partially resistant and susceptible genotypes.

Upon inoculation with *D. pinodella*, the genes *PsOX11*, *PsAPX1, PsCHS3*, and *PsOPR1* showed clear induction in the partially resistant genotype PBA Wharton (**Figure 5B**). Interestingly there was very little induction of all the studied defense related genes except *Pshmm6* in the other partially resistant genotype 10HP249-11HO-7. In the genotype PBA Wharton, a gradual induction of *PsOX11* was observed at 72 HPI (52 fold) and peaked at 96 HPI (114 fold). *PsCHS3* (70 fold) and *PsOPR1* (172 fold) also showed a peak relative mRNA level in PBA Wharton at 96 HPI, while *PsAPX1* showed rapid significant induction at 24 HPI (83 fold) and 96 HPI (57 fold) compared to the expression in susceptible genotypes OZP1604 and WAPEA2211. There was no difference in the expression of *Pshmm6* gene either in partially resistant or susceptible genotypes upon inoculation with *D. pinodella*.

## Discussion

The ascochyta blight disease complex poses a continuous threat to the production of field pea worldwide. Understanding the resistance mechanisms initiated in field peas upon encountering the ascochyta blight pathogens will provide improved strategies to breed new genotypes that can effectively minimize yield loss. Despite previous efforts of characterizing of resistance responses of field pea against the pathogens *P. pinodes* (Fondevilla et al., 2011; Carrillo et al., 2013) and *A. koolunga* (Tran et al., 2018), these processes are still not completely understood. The polygenic nature of resistance in field peas against ascochyta blight indicates that there are complexities in understanding the associated mechanisms. The present study aimed at dissecting the resistance reactions in field pea genotypes inoculated separately with *P. pinodes* and *D. pinodella* using phenotypic, histochemical and molecular approaches.

The expression of five defense related genes, namely, *PsOXII*, *PsAPX1*, *Pshmm6*, *PsCHS3*, and *PsOPR1*, were studied to understand their role in providing resistance against necrotrophic pathogens that cause ascochyta blight in field pea. The genes selected for this study were from different classes such as the peroxidase superfamily (*PsOXII* and *PsAPX1*), the flavonoid and pisatin biosynthesis pathway (*PsCHS3* and *Pshmm6*), and the JA biosynthesis pathway (*PsOPR1*). In previous findings efforts were made to study the expression of these defense related genes against *P. pinodes* in leaf (Fondevilla et al., 2011; Tran et al., 2018) and against *A. koolunga* in the leaves and stems (Tran et al., 2018). In our study, an attempt was made to study the expression of these defense related genes and decipher their role in providing resistance against *P. pinodes* and *D. pinodella* in control and infected, resistant and susceptible lines.

Overall, changes in gene expression were much stronger for *PsOXII, Pshmm6* and *PsCHS3* upon inoculation with the more aggressive *P. pinodes* compared to the less aggressive *D. pinodella*. These expression trends were in parallel to phenotypic assessments that showed that *P. pinodes* was 200% more aggressive in terms of disease severity and spread compared to *D. pinodella.* Similarly, (Hanssen et al., 2011) demonstrated a similar finding when the aggressive isolates of a pepino mosaic virus isolate against tomato seedlings elicited a stronger defense response than milder forms of the pathogen.

*PsOXII* codes for an extracellular enzyme while *PsAPX1* codes for an intracellular enzyme and these belong to class III and class I of the plant peroxidase superfamily, respectively. They play critical roles in plant defense by contributing to the formation of defense barriers (Jiang et al., 2019). Apart from plant defense, class III peroxidases are involved in physiological processes such as formation of lignin (Warinowski et al., 2016), auxin metabolism (Zhang et al., 2014), seed germination (Singh et al., 2015) and aging (Chen et al., 2020). Intrigued by the differential expression of *PsOXII* and *PsAPX1* in field pea against *A. koolunga* (Tran et al., 2018) the same two genes were evaluated against *P. pinodes* and *D. pinodella*. *PsOXII* showed an elevated expression of 170-200 fold in partially resistant genotypes upon inoculation with *P. pinodes*, whereas an inoculation with *D. pinodella* resulted in a 30-85 fold increase in expression in PBA Wharton. These results show that this gene is expressed more in an interaction with the aggressive pathogen *P. pinodes* both in partially resistant and susceptible genotypes although sooner in partially resistant genotypes. This gene expression was noticeably upregulated only in PBA Wharton when inoculated with the less aggressive pathogen *D. pinodella.*

*PsAPX1* had similar patterns of induction in both resistant and susceptible genotypes when inoculated with *P. pinodes*, although induction was slightly higher in susceptible genotypes. Conversely, expression in the *D. pinodella* interaction was very low in three genotypes, but much higher in PBA Wharton. This suggests that on the one hand, APX1 plays role in a susceptible interaction with *P. pinodes*, but not with the more benign *D. pinodella.* This gene also appears to have a different role in PBA Wharton when challenged with this benign pathogen, and this is different than the other resistant line.

Similar elevated expression levels of five peroxidase genes have been previously demonstrated against treatment of *P. pinodes* elicitor (Kawahara et al., 2006) confirming the role of *PsOXII* in the pea and *P. pinodes* interaction. Similar differential response was demonstrated for *PsOXII* and *PsAPX1* where both the genes showed an elevated expression in resistant genotype against an inoculation with *A. koolunga* although the expression was more than 10 times higher in *PsAPX1* compared to *PsOXII*. (Tran et al., 2018). Although it indicates from this study and the previous studies that different resistance mechanisms exists against *P. pinodes, D. pinodella and A. koolunga*, further research with more genes would help confirm these results.

Peroxidases (POD) are a class of proteins that are induced in various biotic stresses (Sasaki et al., 2004). They play an important role in scavenging the excess H_2_O_2_ to maintain the ROS homeostasis in the cell (Ozyigit et al., 2016) and may have played a crucial role in providing partial resistance to the genotypes against the two pathogens under investigation. The leaves of partially resistant genotypes showed significantly fewer intensely stained lesions that was due to decreased cell death and lower generation of H_2_O_2_ compared to the leaves of susceptible plants. This was similar to the work in tomato that showed increased necrotic lesions, more intensely stained leaves, and lower activities of peroxidase enzymes in more susceptible mutants compared to the wild-type plants (Hong et al., 2019). The lack of an efficient scavenging mechanism may result in excessive generation of H_2_O_2_ and can cause oxidative stress resulting in chloroplast and peroxisome autophagy and triggering cell death (Smirnoff and Arnaud, 2019). The presence of antioxidant systems in plants help to eliminate excess H_2_O_2_ generated and thus maintains H_2_O_2_ levels in a normal dynamic balance (Quan et al., 2008). This could result in a lower detection of H_2_O_2_ in the partially resistant genotypes. Apart from the role of cellular signaling, ROS directly kills the pathogen and plays a key defensive strategy during pathogen attack (Paiva and Bozza, 2014). The results obtained in histochemical staining of the leaf samples and rapid induction in the expression of *PsOXII* and *PsAPX1* genes post inoculation with *P. pinodes* and *D. pinodella* confirmed that the association of elevated gene expression and low cell death in partially resistant genotypes compared to the susceptible counterparts. The lower accumulation of H_2_O_2_ in partially resistant genotypes may be due to the efficient scavenging mechanism by these peroxidase genes in comparison to susceptible genotypes. The result obtained in this study are in line with the findings that showed the removal of excessive H_2_O_2_ and limiting the damage caused during an interaction of wheat with *Pyricularia oryzae* ultimately provided greater resistance to the blast disease (Debona et al., 2012). More specifically, during the *D. pinodella* infection resulted an elevated the expression of *PsAPX1* gene which has played a key role to restrict the spread of the pathogen and this gene also played an important role in scavenging the excessive H_2_O_2_ and formed a part of defense reaction. In plants and algae *APX* enzyme catalyze the reduction of H_2_O_2_ and prevents the H_2_O_2_^-^ mediated damage to cells and organs (Ozyigit et al., 2016).

*Pshmm6* and *PsCHS3* encode for enzymes in the field pea isoflavonoid phytoalexin pisatin biosynthesis pathway (Liu et al., 2006). This phytoalexin has played a critical role in initiating defense responses upon inoculation with *P. pinodes* (Fondevilla et al., 2011) and *A. koolunga* (Tran et al., 2018) and the reduced ability to produced pisatin resulted in lower resistance to fungal infection (Wu and VanEtten, 2004). In this study the *Pshmm6* gene had the highest expression levels in partially resistant genotypes when challenged against the aggressive pathogen *P. pinodes* particularly at the later stages of infection (~ 260 – 345 fold) compared to susceptible genotypes (~ 55 – 83 fold). It is clear that the induction levels were relatively low and at equal levels in partially resistant and susceptible field pea genotypes after inoculation with less virulent pathogen *D. pinodella* (~ 21 - 45 fold). This could be due to the presence of two highly conserved *hmm* genes which share 95.8% amino acid identity in field pea (Wu et al., 1997) where the other *hmm* gene may have played a role in initiating a defense response and providing resistance against *D. pinodella*. Further research is needed to decipher the role of these two *hmm* genes in providing resistance against the pathogens.

*PsCHS3* showed greater induction up to ~ 319 fold late in the infection process in the partially resistant genotype PBA Wharton and *P. pinodes* interaction. Similarly low expression levels were observed in other partially resistant genotype (128 fold) and susceptible genotype OZP1604 (~ 120 fold) when inoculated with *P. pinodes*. In PBA Wharton and *D. pinodella* interaction the partially resistant genotype showed high expression level albeit late in infection. The lower disease severity in the partially resistant genotype PBA Wharton provides evidence that strong induction of *Pshmm6* and *PsCHS3* contributed to restrict the growth and spread of both the pathogens especially at later stage of infection. In a similar study in cotton, the knockdown of *GhCH3* gene resulted in the increased susceptibility to the *Verticillium dahliae* infection (Lei et al., 2018), which makes it clear that *GhCH3* gene plays a critical role in providing resistance against *V*. *dahliae*.

The hormone jasmonate has been shown to be involved in plant resistance against necrotrophic pathogens (Veronese et al., 2004). *AtOPR1* encodes for a 12-Oxophytodienoate reductase enzyme in the JA biosynthesis pathway in Arabidopsis (Biesgen and Weiler, 1999). In our studies *PsOPR1* showed a 153 – 229 fold induction at early stage of infection in both partially resistant and susceptible plants when inoculated with *P. pinodes* although the expression was significantly higher in the susceptible plants. These results were in agreement with that obtained by Fondevilla et al. (2011) where *OPR1* was shown to have high induction in susceptible genotype Messire and no induction in the resistant genotype P665 upon inoculation with *P. pinodes*. Interestingly there was clear high and gradual induction in partially resistant genotype PBA Warton up to 156 – 172 fold albeit late in the infection compared to its susceptible counterparts, when inoculated with *D. pinodella*. High induction of *PsOPR1* may not be enough to counter the aggressive pathogen like *P. pinodes* while a similar level of induction was sufficient enough to provide resistance against *D. pinodella*.

The visual quantification of damaged leaf area due to pathogen infection can be a challenging task due to its subjective nature. In recent times sensor based approaches have been widely used to assess the leaf damage post pathogen infection. Application of high throughput image processing techniques has enabled us to quantify the spread of the infection by *P. pinodes* and *D. pinodella*. The image processing techniques helped in the estimation of damaged leaf area due to infection and showed a 4.9 times higher detection of generated H_2_O_2_ in susceptible plants inoculated with *P. pinodes* than *D. pinodella*. Similar image processing techniques have been deployed to detect bacterial and fungal diseases in bean leaf (Singh and Misra, 2017) and estimation of the disease spread (Bock et al., 2010). Image analysis not only helps in detecting disease symptoms but also provides an enhanced ability to differentiate the genotypes with varying disease severity. In this regard our findings confirm those of another study investigating bacterial blight in bean (Xie et al., 2012). The results obtained by digital image analysis play a pivotal role in accurately phenotyping disease severity for detailed genetic analysis. This technique has been used as a tool in identifying quantitative trait loci for powdery mildew resistance in lettuce (Simko et al., 2014). The strong correlation between leaf damage digital area and disease severity shows that the value of using digital image analysis as a surrogate method in assessing disease severity.

The disease severity scores of experiment 1 and leaf damage digital area of experiment 2 inoculated against *P. pinodes* and *D. pinodella* showed high positive correlations, validating the disease assay and highlighting the value of the imaging technology. Furthermore, the reliability of the assay allows the selection of genotypes across different experiments and provides confidence in selecting improved lines for disease resistance breeding.

## Conclusion

A range of field pea genotypes were evaluated to characterization the resistance against the two pathogens *P. pinodes* and *D. Pinodella* through phenotypic, histochemical and molecular approaches. Among the two pathogens *P. pinodes* was more aggressive compared to *D. pinodella*, exhibited a clear differential disease severity between genotypes against the two pathogen. The breeding lines 11HP-302-12HO-1 and 10HP249-11HO-7 showed lower disease severity and less accumulation of H_2_O_2_ against individual pathogens. The partially resistant genotype 11HP-302-12HO-1 showed an elevated early expression of *PsOXII*, late induction of *Pshmm6* to *P. pinodes*. Along with the breeding line PBA Wharton showed late expression of *PsCHS3* gene against *P. pinodes* and demonstrated high expression of *PsPOXII, PsAPX1, PsCHS3*, and *PsOPR1* against milder pathogen *D. pinodella* indicating that the resistance is multifaceted. The variation in responses exhibited against different pathogens of ascochyta blight can be harnessed through a recurrent selection breeding programs by combining different sources of partial resistance as identified in this work. The high correlation between data from two independent experiments show the stability of genotypes and these partially resistant breeding lines can be effectively used in disease resistance breeding to develop varieties that produce sustainable yield by overcoming this disease complex.

## Data availability statement

The original contributions presented in the study are included in the article/**Supplementary Material**, further inquiries can be directed to the corresponding author/s.

## Author contributions

SJ and GR conceived the experiment. SJ designed, conducted, performed statistical analysis and wrote the draft. GR supported the study. BP contributed scientific inputs. All authors edited the manuscript. All authors contributed to the article and approved the submitted version.

## Funding

This study was made possible by joint collaboration by Agriculture Victoria Research and Grain Research Development Corporation through the research project DAV1607 - 010BLX PulseBio Project 3.

## Conflict of interest

This study received funding from Grain Research Development Corporation. The funder was not involved in the study design, collection, analysis, interpretation of data, the writing of this article or the decision to submit it for publication. All authors declare no other competing interests.

## Publisher’s note

All claims expressed in this article are solely those of the authors and do not necessarily represent those of their affiliated organizations, or those of the publisher, the editors and the reviewers. Any product that may be evaluated in this article, or claim that may be made by its manufacturer, is not guaranteed or endorsed by the publisher.
