# Supplementary material for: Characterization of field pea (Pisum sativum) resistance against Peyronellaea pinodes and Didymella pinodella that cause ascochyta blight
Source: Front Plant Sci. 2022 Oct 24;13:976375. doi: 10.3389/fpls.2022.976375 (PMC9637924; doi:10.3389/fpls.2022.976375)
Supplement: Supplementary file 1 [file Presentation_1.pptx]

## Slide 1
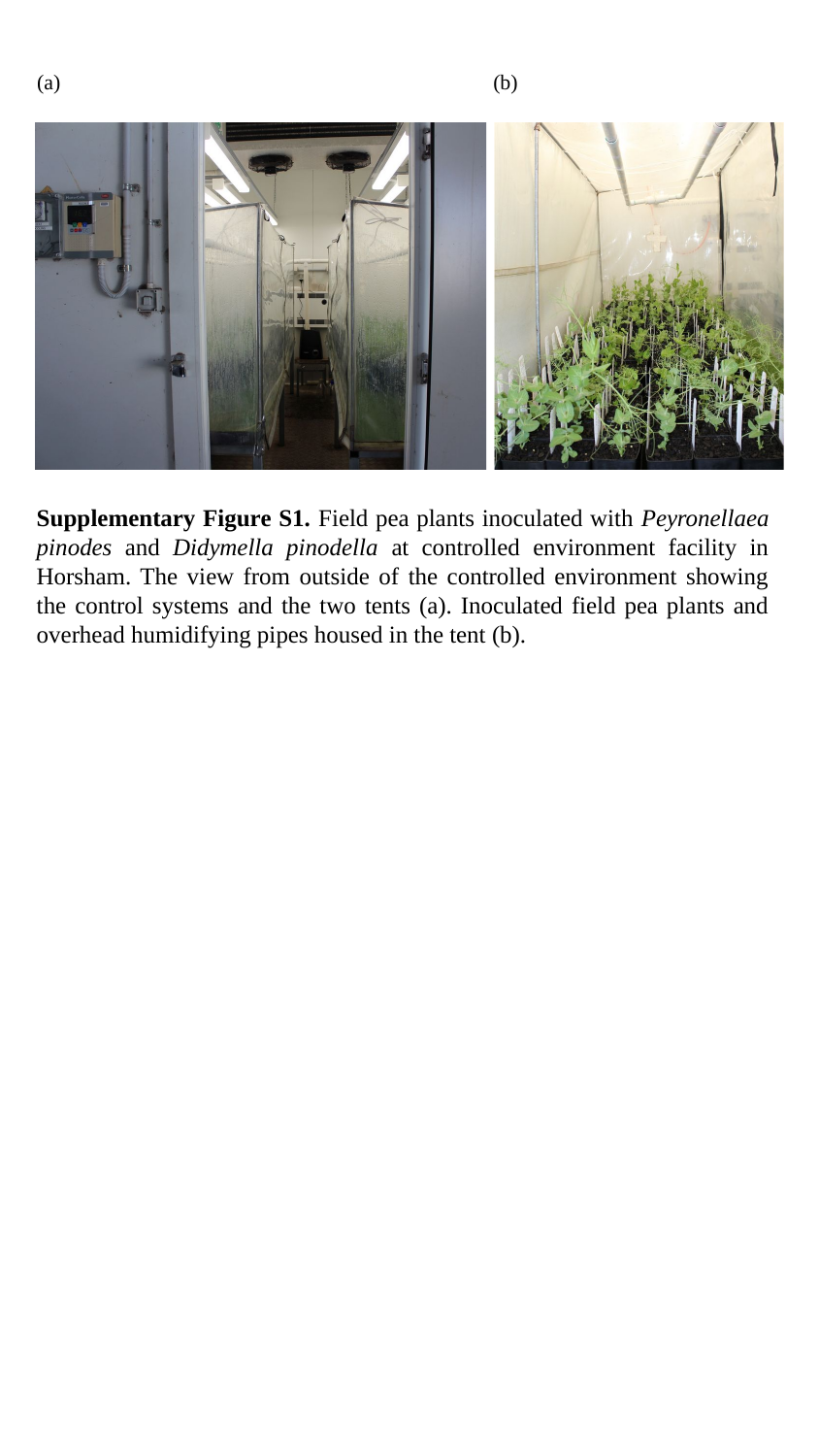

(a)
(b)
Supplementary Figure S1. Field pea plants inoculated with Peyronellaea pinodes and Didymella pinodella at controlled environment facility in Horsham. The view from outside of the controlled environment showing the control systems and the two tents (a). Inoculated field pea plants and overhead humidifying pipes housed in the tent (b).

## Slide 2
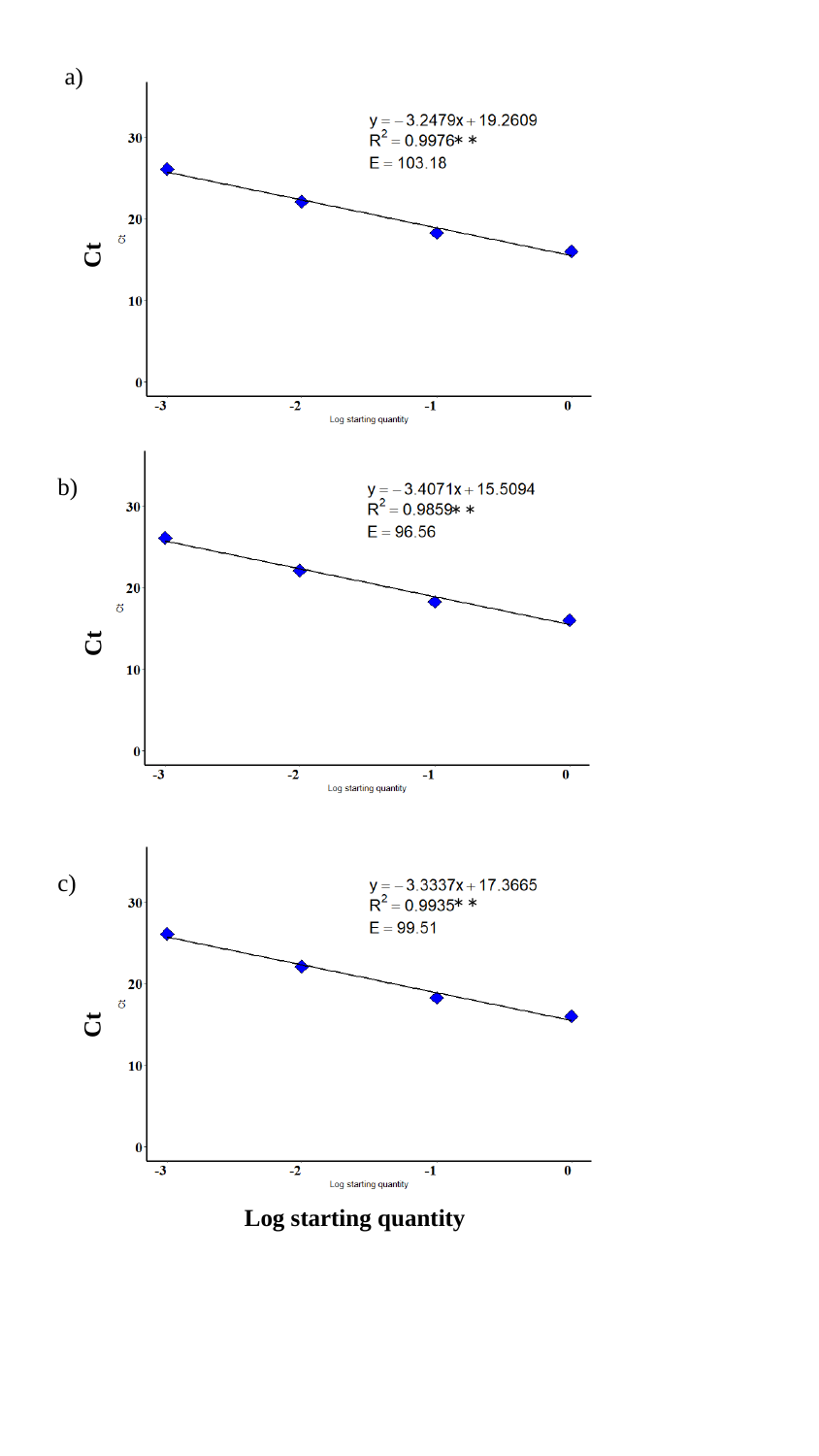

a)
**
Ct
b)
**
Ct
c)
**
Ct
Log starting quantity

## Slide 3
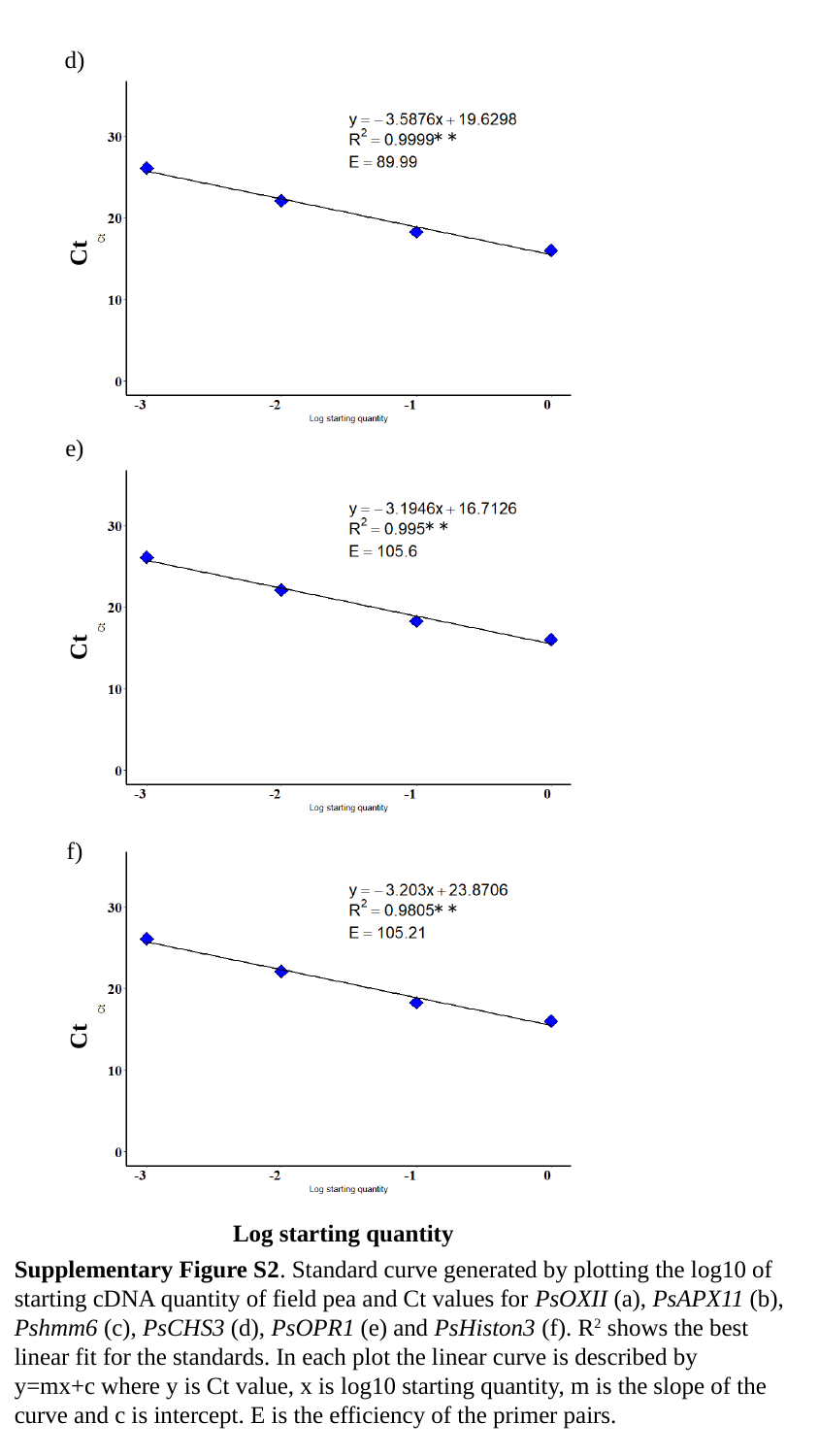

d)
**
Ct
e)
**
Ct
f)
**
Ct
Log starting quantity
Supplementary Figure S2. Standard curve generated by plotting the log10 of starting cDNA quantity of field pea and Ct values for PsOXII (a), PsAPX11 (b), Pshmm6 (c), PsCHS3 (d), PsOPR1 (e) and PsHiston3 (f). R2 shows the best linear fit for the standards. In each plot the linear curve is described by y=mx+c where y is Ct value, x is log10 starting quantity, m is the slope of the curve and c is intercept. E is the efficiency of the primer pairs.
